# Inferior Vena Cava Thrombus Associated With Renal Cell Carcinoma With an Atypical Radiological Presentation: A Case Report

**DOI:** 10.7759/cureus.45193

**Published:** 2023-09-13

**Authors:** Kareem Elgendi, Mohamadhusni Zarli, Sohaib Ahmed, Nicole Szell

**Affiliations:** 1 Dr. Kiran C. Patel College of Osteopathic Medicine, Nova Southeastern University, Clearwater, USA; 2 Dr. Kiran C. Patel College of Osteopathic Medicine, Nova Southeastern University, Fort Lauderdale, USA; 3 Urology, Advanced Urology Institute, Clearwater, USA

**Keywords:** tumor burden, renal masses, ivc thrombus, venous tumor thrombus, renal cell carcinoma

## Abstract

Renal cell carcinoma (RCC) is characterized by the development of kidney masses, which can lead to various long-term complications. Among the extrarenal manifestations associated with RCC, the formation of a thrombus within the inferior vena cava (IVC) is particularly prevalent due to the substantial tumor burden imposed by the kidneys. In this report, we present an exceptional case involving an 80-year-old male patient who presented with an intravascular thrombus within the inferior vena cava (IVC), which originated from RCC. The diagnosis of RCC was conclusively established through core needle biopsy and subsequent tumor marker staining. Remarkably, despite the confirmation of RCC within the IVC thrombus through biopsy and tumor marker analysis, radiological assessments failed to reveal any discernible renal cell masses within the kidneys. The patient subsequently received treatment for RCC with a combination regimen of cabozantinib and nivolumab, which resulted in a noteworthy improvement in his clinical condition. The presentation of RCC in this report is notably atypical, given that the biopsy of the thrombus yielded definitive evidence of RCC while radiological investigations did not yield any indications of renal masses or a tumor burden within the kidneys that would typically be associated with RCC.

## Introduction

Renal cell carcinoma (RCC) is the most prevalent form of kidney cancer and is more common in men than women, with the peak incidence occurring in the sixth and seventh decades of life [1]. One of the critical characteristics of RCC is its tendency to invade the renal vein and inferior vena cava (IVC), leading to the formation of venous tumor thrombus [2]. Intravascular tumor growth along the renal vein into the inferior vena cava (IVC) occurs in up to 10% of all patients with RCC [3], and further extension of the tumor reaching the right atrium (RA) is detected in approximately 1% of all patients. This tumor thrombus is a significant predictor of adverse outcomes in RCC patients, and the level of thrombus is an independent predictor of survival [2]. While RCC may present with flank pain, hematuria, and an abdominal mass, this classical triad is only present in a small percentage of cases [1]. Our case report is unique due to the rare presentation of tumor thrombus, extending from the right renal vein into the IVC and right femoral vein, without any evidence of renal mass or malignancy. This is a distinct presentation that lacks prior documentation in the available literature.

## Case presentation

The patient in this case was an 80-year-old male with a medical history that included hypertension, dyslipidemia, type 2 diabetes mellitus, aortic stenosis, chronic congestive heart failure, and a recent pulmonary embolism. He initially presented to the hospital with increasing fatigue, weakness, chest discomfort, and back pain and was found to be hypotensive and in renal failure. Further evaluation revealed an elevated creatinine level of 1.8 mg/dL (baseline of 1.4 mg/dL), hyperkalemia of 7.0 mmol/L, and increased lactate dehydrogenase (LDH) levels of 3.4 mmol/L. Blood urea nitrogen (BUN) was elevated at 35 mg/dL. AST and ALT levels were also elevated at 130 IU/L and 136 U/L, respectively. WBC count was increased at 19.2 th/uL and hemoglobin and hematocrit (H&H) were low at 11.1 g/dL and 35.6%, respectively.

Physical exam on presentation was unremarkable. The patient went on to develop progressive edema in the abdomen, genitalia, and lower extremities, which was attributed to the occlusion of the inferior vena cava (IVC). He did not meet the criteria for nephrotic syndrome. At this point, Eliquis was discontinued and IV heparin was initiated. The patient was evaluated by both nephrology and infectious disease specialists.

**Figure 1:**
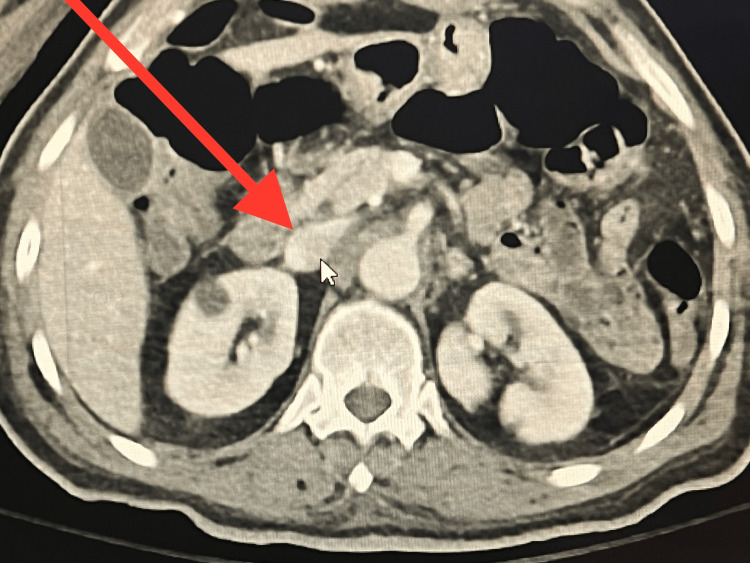
Abdominal CT scan showing distention of the right renal vein and significant distention of the suprarenal vena cava.

CT imaging of the patient's abdomen and pelvis (Figure 1) showed distention of the right renal vein and significant distention of the suprarenal vena cava, which could have been due to either a thrombus or an underlying mass. The radiologist’s report noted non-hydronephrotic kidneys with stranding regional to the right kidney and distention of the right renal vein as well as significant distention of the suprarenal vena cava extending up to its junction with the right atrium. An underlying thrombus or mass was suspected. No discrete renal masses were noted within the kidneys.

**Figure 2:**
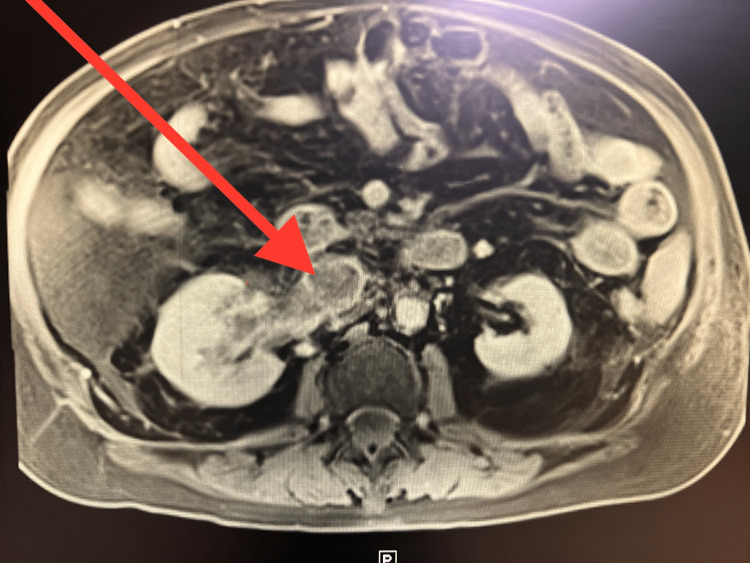
MRI abdomen revealing a filling defect within the right renal vein.

A subsequent MRI of the abdomen (Figure 2) revealed a filling defect within the right renal vein that extended into the inferior vena cava (IVC), indicating the presence of a blood clot or tumor. The radiologist’s report noted that both kidneys demonstrated a symmetrical enhancement with a filling defect identified within the right renal vein, which extended into the IVC and showed distention. This could’ve represented changes due to either a blood clot versus a tumor thrombus. Interventional radiology (IR) venography of the IVC was performed following a core biopsy, and the surgical pathology report suggested totally necrotic renal cell carcinoma with complete occlusion due to extensive thrombus.

Given the risk of surgical complications, systemic therapy with cabozantinib and nivolumab was initiated. The patient showed gradual improvement, with a decrease in lower extremity edema. The patient was eventually discharged and was scheduled to complete an outpatient positron emission tomography (PET) scan as well as a follow-up with a new oncology team in his home state.

## Discussion

Renal cell carcinoma (RCC) is a type of kidney cancer that arises from the renal cortex and is the most prevalent form of kidney cancer worldwide. Venous tumor thrombus is a unique characteristic of renal cell carcinoma. It has been reported to occur in 4%-10% of renal neoplasms [4]. Venous tumor thrombus in renal cell carcinoma can occur in several locations with a reported prevalence of 10%-18% in the renal vein, 4%-23% in the IVC, and 1% extending into the right atrium [2]. Tumor extension into the IVC is found more commonly in right-sided than left-sided renal cell carcinomas and typically invades intra-luminally without involving the wall of the vena cava [5]. Many classification systems have been established for the extent of IVC thrombus in RCC. The Mayo Clinic classification divides it into four levels: level 1 is for a thrombus that involves <2 cm above the renal vein; level 2 is for an extension more than 2 cm but below the intra-hepatic vena cava; a level 3 thrombus reaches the intra-hepatic portion of the vena cava; and level 4 is above the diaphragm [6]. In a congenitally normal-appearing IVC, thrombosis is usually caused by abdominal masses such as renal cell carcinomas, pancreatic carcinomas, large uterine fibroids, Budd-Chiari syndrome, liver abscesses, retroperitoneal masses, abdominal aortic aneurysms, etc. IVC thrombus formation in such conditions is attributed to disturbances in Virchow’s triad due to compressive forces exerted by the tumor masses toward adjacent structures [7]. The presentation of IVC thrombosis can be ambiguous and symptoms directly attributable to tumor thrombus are rare. Therefore, diagnosis of IVC thrombus is generally detected from radiologic imaging, which is highly sensitive in diagnosing these conditions [3].

The patient initially presented with nonspecific symptoms suggestive of pulmonary/cardiovascular compromise. Abdominal imaging revealed the presence of an IVC thrombus-associated renal cell carcinoma (RCC), which was subsequently confirmed through a core needle biopsy of the IVC mass/thrombus. Surgical pathology with immunoperoxidase stains for CD-10, RC, PAX-8, and pankeratin (renal cell and epithelial tumor markers) showed positive staining in all four preparations, confirming the diagnosis of renal cell carcinoma. However, there were no identified masses within the renal pelvis or kidney on imaging that could explain the presence of tumor burden or clot formation. This presentation is distinct and currently lacks any prior documentation in the available literature. This case underscores a noteworthy clinical scenario wherein a biopsy-confirmed renal cell carcinoma-associated thrombus lacked corresponding radiological evidence of a primary renal tumor. This atypical presentation deviates from the conventional understanding in current literature that such thrombi typically emanate from the renal parenchyma, readily discernible through imaging modalities. Consequently, our case contributes to the existing body of literature by emphasizing the imperative of considering renal cell carcinoma in symptomatic patients who exhibit no radiological manifestations of renal tumors.

In this case, therapeutic intervention for renal cell carcinoma commenced with a regimen comprising cabozantinib and nivolumab. The collaborative decision of the medical team favored this pharmacological approach over surgical intervention, additionally incorporating anticoagulation therapy with enoxaparin. Notably, treatment with cabozantinib and nivolumab yielded substantial improvements in the patient's clinical status. Consequently, the patient has been scheduled for follow-up consultations with an oncology team in their home state to perpetuate systemic management for renal cell carcinoma. Nevertheless, the lingering inquiry pertains to the genesis of renal cell carcinoma in this context and its relationship with the development of the inferior vena cava thrombus, given the conspicuous absence of any radiological evidence indicative of renal mass or malignancy.

## Conclusions

The case presented provides evidence of an inferior vena cava (IVC) thrombus in the context of renal cell carcinoma, confirmed via core needle biopsy, despite the absence of observable masses or indications of renal cell carcinoma (RCC) on renal imaging. This case highlights an uncommon manifestation of renal cell carcinoma, wherein a thrombus develops within the IVC without detectable renal involvement. This presentation is distinct and lacks prior documentation in the available literature. This case adds to the current literature by emphasizing the importance of considering renal cell carcinoma as a potential etiology in the presence of an IVC thrombus, even in the absence of radiologic evidence of renal masses or malignancy within the kidneys.
